# Relationships Between Metabolism of Cryopreserved Equine Sperm Determined by the Seahorse Analyzer and Sperm Characteristics Measured by Flow Cytometry and Computer-Assisted Analysis of Motility

**DOI:** 10.3390/vetsci12121109

**Published:** 2025-11-21

**Authors:** Fokko Mathias Strassner, Lukas Demattio, Mathias Siuda, Eleni Malama, Gérard Muffels, Heinrich Bollwein

**Affiliations:** 1 Clinic of Reproductive Medicine, Department for Farm Animals, Vetsuisse Faculty, University of Zurich, Winterthurerstrasse 260, CH-8057 Zurich, Switzerland; fokkomathias.strassner@uzh.ch (F.M.S.); msiuda@vetclinics.uzh.ch (M.S.); emalama@vetclinincs.uzh.ch (E.M.); 2 AgroVet-Strickhof, Eschikon 27, CH-8315 Lindau, Switzerland; 3 Holsteiner Verband, Westerstr 93, 25336 Elmshorn, Germany; hengststall@holsteiner-verband.de

**Keywords:** stallion spermatozoa, metabolism, Seahorse Analyzer, cryopreservation

## Abstract

Cryopreservation of stallion spermatozoa remains a challenge in equine reproduction, as it induces mitochondrial dysfunction that cannot be fully captured by conventional sperm quality assessment and requires further investigation. This study provides a detailed bioenergetic evaluation of cryopreserved stallion sperm using Seahorse XFp technology to measure basal oxygen consumption rate (OCR) and to perform a MitoStress Test. Three ejaculates from 20 Warmblood stallions (licensed by the Holsteiner Verband, Elmshorn, Germany) were analyzed at 15 min intervals from 15 to 120 min after thawing. CASA and multiparametric flow cytometry were used to assess motility, sperm viability, reactive oxygen species (ROS) synthesis, lipid peroxidation (LPO), and DNA fragmentation index (%DFI). Seahorse analysis revealed marked intra- and inter-individual variability in basal OCR among frozen–thawed samples. Functional mitochondrial parameters showed only partial correlations with motility and viability results, underlining the added diagnostic value of combined metabolic profiling. Over the 2 h measurement, viability and motility displayed an increasingly negative correlation with ROS synthesis, while no significant relation was found between OCR and ROS synthesis or LPO. These findings demonstrate that stallion sperm mitochondria are highly sensitive organelles whose functional state cannot be fully assessed by routine motility or viability testing alone. Integrating Seahorse-based profiling with CASA and flow cytometry can improve sperm quality assessment. Moreover, this novel approach has been proven to be a valuable tool, offering broad opportunities for further research in stallion reproduction.

## Simple Summary

Cryopreservation of stallion semen is an essential technique for breeding and genetic exchange in horses worldwide. However, frozen–thawed sperm generally show reduced quality compared to fresh semen, as cryoinjuries particularly affect the mitochondrial membranes. In this study, we examined the metabolism of cryopreserved semen from 20 Warmblood stallions to assess mitochondrial function and its relationship to conventional sperm quality parameters such as motility, viability, oxidative stress, and DNA integrity. We found considerable differences not only between individual stallions but also between different ejaculates within stallions. Our results show that assessing mitochondrial function provides valuable additional information about sperm quality. This new approach can improve standard sperm evaluation and facilitates further research in this field.

## 1. Introduction

For equine reproduction, cryopreserved stallion sperm is becoming increasingly important as it is the only method available for long-term sperm storage. This technology enables the global distribution of valuable genetics and allows retired stallions to continue contributing to reproduction [1,2]. Consequently, although liquid-preserved semen is still used far more frequently in practice [3], cryopreserved semen has substantial economic significance. However, compared to liquid preservation, cryopreservation causes more sperm damage that is not entirely detected by conventional quality assessment, such as analyses of motility or viability [4]. This highlights the great importance of advancing and integrating more detailed approaches to sperm quality assessment.

Various sperm functions must be intact to successfully fertilize an oocyte, resulting in the development of an embryo [5]. For this reason, different parameters of sperm evaluation are combined to improve the reliability of fertility prognosis [6,7,8]. To achieve the most efficient combination of different sperm parameters, it is important to consider the correlation between them. High correlations reduce the capability of a combined model to predict fertility. Intra-individual variations indicate necessity of examining each ejaculate of a stallion to draw conclusions about its fertility [9].

Cryopreservation, among other effects, gives rise to oxidative stress in spermatozoa during freezing and thawing [10]. Thawing induces osmotic stress, which damages particularly the mitochondrial membrane. This cryodamage leads to metabolic degradation of sperm and increases release of reactive oxygen species (ROS). Oxidative stress can then result in the peroxidation of DNA, proteins, and lipids. [11,12].

Mitochondria are highly specialized and sensitive organelles that play a crucial role in regulating sperm function and, ultimately, fertilization success. Unlike mitochondria in somatic cells, sperm mitochondria are uniquely adapted to control a range of tightly coordinated processes, including progressive motility, hyperactivation, capacitation, acrosome reaction, and the final sperm–oocyte fusion [13]. Given this functional complexity, a comprehensive assessment of sperm quality requires a multiparametric approach that combines structural, kinetic, and metabolic parameters.

The metabolism of stallion sperm bases mainly on oxidative phosphorylation (OXPHOS), which is the major source of ATP generation [14,15,16]. OXPHOS therefore plays a key role for fertility-relevant parameters such as sperm motility and membrane integrity [17]. Metabolism was typically examined by assessing the mitochondrial membrane potential (MMP) using fluorescent cationic dyes, fluorescence microscopy, and flow cytometry [13]. The dye JC-1 is widely used. It emits green light in sperm with low MMP and orange light in sperm with high MMP [18]. JC-1 is very sensitive and specific, but it requires two detectors and therefore renders its use in multicolor assays [19] rather elaborate. Fluorochromes such as MitoTracker™ Deep Red or Mitoprobe™ DiIC_1_(5), which generate more or less intense fluorescence, depending on the membrane potential, can be integrated well in multicolor assays. These MMP tests do not provide much bioenergetic information [13] and are only qualitatively reliable [20]. Another approach to directly assess metabolism is to measure the oxygen consumption of sperm, which was already performed for stallion semen [21,22].

The Seahorse XF Analyzer (Agilent Technologies, Inc., Santa Clara, CA, USA) is used to measure the oxygen consumption rate (OCR) of cells, which corresponds to OXPHOS, and the extracellular acidification rate (ECAR), which represents glycolysis [23]. Programmed addition of mitochondrial effector drugs during the assay allows detailed analysis of the main cellular metabolic pathway. To date, studies have been performed on sperm of mice, men, bulls, boar, and stallion [23,24,25,26,27,28,29,30,31,32,33]. Research in bulls has revealed that highly motile sperm exhibit a significantly elevated metabolic rate and ATP production compared to less motile sperm, with additional evidence indicating breed-specific differences in metabolic pathways [27]. Moraes et al. [31] noticed that cryopreserved bull sperm have an increased non-mitochondrial metabolism and basal oxygen consumption compared to fresh semen, which is probably due to freezing-induced mitochondrial damage. The same study revealed a negative correlation between proton gap and fertility. Algieri et al. [30] demonstrated the adaptability of bovine sperm. Depending on the concentration of glucose in the surrounding medium, sperm of this species performed either glycolysis or OXPHOS to gain ATP. Stallion sperm differ in their metabolism from bovine as they exclusively perform OXPHOS [17]. A study published recently examined fresh stallion sperm using the Seahorse Analyzer. The results of this investigation confirmed that a major part of ATP is produced via OXPHOS [33]. However, cryopreserved stallion sperm has not been examined with the Seahorse Analyzer to date, and data on within and between stallion variabilities in equine sperm assessed with this method are still lacking.

The aim of this study was to examine frozen–thawed stallion sperm using the Seahorse Analyzer, paying particular attention to intra- and inter-individual differences in the OCR and ECAR. Furthermore, the results were correlated with other established sperm quality assays to determine whether this method provides additional information on sperm quality.

## 2. Materials and Methods

### 2.1. Chemicals and Reagents

The chemicals NaCl, MgSO_4_, KH_2_PO_4_, Na-Lactate, and bovine serum albumin (BSA) utilized for the preparation of Tyrode’s solution, phosphate-buffered saline (PBS), Triton, and the fluorochromes PI, and Dihydroethidium (HE) were purchased from Sigma-Aldrich Co. (Buchs, Switzerland). The remaining chemicals KCl, CaCl_2_, Hepes buffer, and penicillin for the preparation of Tyrode’s solution and all chemicals for the SCSA™ except Triton and Acridine Orange were purchased from Carl Roth GmbH + Co KG (Karlsruhe, Germany). Acridine Orange was obtained from Polysciences Europe GmbH (Eppelheim, Germany). Bodipy™ 493/503 (BP), MitoProbe™ DiIC_1_(5), Fluo-4 AM, and Calcein Violet were obtained from Thermo Fisher Scientific Inc. (Waltham, MA, USA). PE-PNA was bought from GeneTex Inc. (Irvine, CA, USA). The reagents XF Calibrant and the XF Cell MitoStress Test kit, which contains the injections of oligomycin, FCCP, and antimycin A/rotenone, were purchased from Agilent Technologies, Inc. (Santa Clara, CA, USA). Gent extender (Ref.:13571/1045, Minitüb GmbH, Tiefenbach, Germany), containing egg yolk, glycerol, and antibiotics, was used to dilute fresh semen prior to cryopreservation.

### 2.2. Preparation of Media

For the preparation of Tyrode’s solution, 96 mM NaCl, 3.1 mM KCl, 2.0 mM CaCl + 2H_2_O, 0.4 mM MgSO_4_ + 7H_2_O, 0.3 mM KH_2_PO_4_, 21 mM Na-Lactate, 20 mM Hepes buffer, 3 mg/mL BSA, and 0.05 mg/mL penicillin were used. The pH level was 7.4 and the osmolality amounts to 320 ± 5 mosmol/kg. Tyrode’s solution was produced weekly, sterile filtered, and warmed to 37 °C before use.

### 2.3. Stallions

For this study, 3 cryopreserved ejaculates from each of 20 (*n* = 60) Warmblood stallions (licensed by the Holsteiner Verband, Elmshorn, Germany), collected from August to February in the years 1996 to 2022, were examined. The stallions were 9.1 ± 5.75 years old (minimum: 3 years; maximum: 22 years) at semen collection. For each stallion, three ejaculates were collected within a median period of 13 days (minimum: 3 days; maximum: 71 days). The stallions represented a random sample of actively breeding sires from the Holsteiner Verband population and were not selected based on specific fertility or performance criteria, except that all were clinically healthy and routinely used in regular breeding service at the time of semen collection.

### 2.4. Semen Collection and Processing

Ejaculates were collected using a prewarmed (41–43 °C) artificial vagina model “Hannover” and examined immediately afterwards macroscopically for color, odor, admixtures, and consistency. Subsequently, ejaculate volume and sperm concentration were determined to calculate the total sperm number per ejaculate. The total and progressive motility were estimated by experienced examiners using a phase-contrast microscope at 100× magnification. Gent extender was added before centrifugation (800× *g* for 10 min) and used to resuspend the pellet to achieve a final concentration of 200 × 10^6^ sperm/mL and 3.5% of glycerol as a cryoprotectant. The sperm were subsequently filled into 0.5 mL straws and equilibrated for 12 min at +4 °C. The straws were frozen using the automatic freezer Mini-Digitcool 1400 (IMV Technologies, L’Aigle, France) at a rate of −10 °C/min down to −25 °C and then at a rate of −25 °C/min down to −140 °C. The frozen sperm was stored in liquid nitrogen (−196 °C).

For the examination, one straw per ejaculate was thawed in a water bath (37 °C for 30 s), opened, and decanted into a preheated 1.5 mL Eppendorf tube (Eppendorf SE, Hamburg, Germany) placed on a warming plate (37 °C).

### 2.5. Computer-Assisted Sperm Analysis

The IVOS II CASA system with software version 1.10.1 (Hamilton Thorne Inc., Beverly, CA, USA) was used for the assessment of sperm motility. A prewarmed (37 °C) 20 µm deep 2-chamber Standard Count Leja^®^ slide (IMV Technologies, L’Aigle, France) was filled with 6 μL of diluted semen to assess in at least 8 randomly chosen fields a minimum of 1000 cells. At a frame rate of 60 Hz, this results in 30 frames per field. Total motility as a percentage was determined in each sample.

### 2.6. Flow Cytometry

#### 2.6.1. Sperm Chromatin Structure Assay

The Sperm Chromatin Structure Assay (SCSA^TM^) was used to determine DNA fragmentation using flow cytometry. Analysis was performed with a CytoFlex Flow Cytometer with a 488 nm laser and CytExpert Software (CytoFlex version 2.4.0.28; Beckmann Coulter Inc., Nyon, Switzerland). The obtained data were analyzed by FCS Express 4 Flow Research Edition (De Novo Software, Pasadena, CA, USA).

The assay was performed as described by Evenson [34]: acidic detergent solution (400 µL) was added to 200 µL of semen diluted with Tris-Na-Cl-EDTA (TNE) solution to a final concentration range of 1–2 × 10^6^ sperm/mL. After mixing for 30 s, 1.2 mL of Acridine Orange staining solution (6.0 µg AO/mL) was added. Flow cytometric analysis was performed exactly 3 min after the addition of staining solution. AO binds to the hydrogen bonds of the DNA and emits green light (filtered through 525/40 nm band-pass filter (BP); FITC channel) in case of double bonds (intact DNA) or red light (filtered through 610/20 nm BP; ECD channel) in case of single bonds (fragmented DNA) after stimulation with a 488 nm laser. For each analysis, 10.000 cells were analyzed at a rate of 200 cells/s. The DNA fragmentation index (%DFI) indicates the percentage of sperm in a sample with fragmented DNA [35].

#### 2.6.2. Multicolor Assay

The CytoFlex Flow Cytometer with the CytExpert Software for CytoFlex version 2.4.0.28 (Beckmann Coulter Inc., Nyon, Switzerland) was used for the multicolor assays. The device had a violet (405 nm) laser with 5 channels, as well as a blue (488 nm) laser with 5 channels and a red (638 nm) laser with 3 channels. The flow rate was set to 60 µL/min and 500–1000 events/s. Per sample, 10,000 cells were examined. A forward scatter area versus forward scatter height density plot was used to identify and exclude doublets, which were then verified by a side scatter area versus side scatter height plot. The gates were set to ensure that only individual cells were measured.

##### Viability and Metabolism

One multicolor assay was performed to examine viability and metabolism as previously described for bovine sperm [36]. The assay contains a panel of 5 fluorochromes to measure intracellular esterase activity (CellTrace™ Calcein Violet AM), assess the plasma membrane integrity (PI), determine the acrosomal status (PE-PNA), the intracellular calcium level (Fluo-4 AM), and the mitochondrial membrane potential (MitoProbe™ DiIC_1_), respectively.

Cell viability can be inferred from high esterase activity. Cells stained with Calcein Violet AM with high esterase activity (C_pos_), excited by a violet laser, emit violet fluorescence (detected with 450/45 nm BP; PB450 channel). The fluorescent dye PI is used to determine the integrity of the plasma membrane. PI can only enter the cell if the plasma membrane is compromised and emits red fluorescence (filtered through 690/50 nm BP; PC5.5 channel) when excited by blue laser. Plasma membrane intact sperm (PI_neg_) emit no fluorescence after excitation. PE-PNA binds to the outer acrosomal membrane. After stimulation with a blue laser, cells with a defective or reacted acrosome emit orange light (detected with 585/42 nm BP; PE channel). Sperm with an intact acrosome (PNA_neg_) show no orange fluorescence after excitation with blue laser. The level of free intracellular Ca^2+^ correlates with the intensity of green fluorescence (filtered through 525/40 nm BP; FITC channel) emitted after staining with Fluo-4 AM and stimulation with the blue laser. High intracellular Ca^2+^ levels (F_pos_) fluoresce high green, while cells with low intracellular Ca^2+^ (F_neg_) emit low green fluorescence after excitation with blue laser. DiLC_1_ is a cationic dye that can differentiate mitochondrial membrane potentials (MMP) in cells. Stimulated by red laser, cells with high MMP (M_pos_) emit a deep red fluorescence, while cells with low MMP fluoresce in red (captured with 660/20 nm BP; APC channel).

For the examination of each sperm sample with the multicolor assay, semen was diluted in a 250 µL well of a 96-well plate at a concentration of 1 × 10^6^ sperm/mL with Tyrode’s solution to a final volume of 244.75 µL. Prior to analysis, the fluorochromes were combined in a master mix with the final concentration per sample of 2 µM Fluo-4 AM (2.5 µL), 0.015 µM DilC1 (0.375 µL), 0.8 mM Calcein Violet AM (0.375 µL), 5 mg/mL PI (1.5 µL), and 1 mg/mL PE-PNA (0.5 µL). These 5.25 µL of master mix were added to each reaction well. The samples were incubated for 15 min at 37 °C in complete darkness before analysis.

The flow cytometric analysis results in several subpopulations, of which the following were of interest: sperm with high intracellular esterase activity (C_pos_), intact plasma membrane (PI_neg_), intact acrosome (PNA_neg_), low intracellular Ca^2+^ (F_neg_), and high MMP (M_pos_). The combined sperm subpopulation C_pos_PI_neg_PNA_neg_F_neg_M_pos_ is defined as “Viability”.

##### Lipid Peroxidation and Reactive Oxygen Species

This assay for the measurement of lipid peroxidation (LPO) and synthesis of reactive oxygen species (ROS) in M_pos_ sperm was performed with CytoFlex Flow Cytometer with the CytExpert Software for CytoFlex version 2.4.0.28 (Beckmann Coulter Inc., Nyon, Switzerland). The protocol used for this study was previously described for bovine semen [37]. Dihydroethidium (HE) was used to determine intracellular ROS production. The generation of superoxide anion, hydrogen peroxide, hydroxyl ion, and peroxynitrite anion can be quantified by measuring the intensity of red fluorescence (filtered through 610/20 nm BP; ECD channel) of HE after oxidation under stimulation with a blue laser. The assay also contained DiIC_1_ to analyze the ROS production in M_pos_ cells. Bodipy™ 581/591 C11 (Bodipy) was used to investigate LPO of the sperm plasma membrane. This fluorochrome binds to the plasma membrane. After its oxidation and excitation by blue laser, there is an increase in green fluorescence (detected by 525/40 nm BP; FITC channel). The combined staining with DiIC_1_ allowed the analysis of LPO in M_pos_ sperm.

To determine LPO and generation of ROS simultaneously in each sample, the semen was diluted in a 250 µL reaction well of a 96-well plate to a concentration of 1 × 10^6^ sperm/mL with Tyrode’s solution to a final volume of 234.625 µL. Prior to analysis, the fluorochromes were combined in a master mix with a final concentration per sample of 0.225 μM HE (7.5 µL), 0.015 µM DilC_1_ (0.375 µL), 10 μM BP (2.5 µL). These 10.375 µL of master mix were added to each reaction well and incubated for 15 min at 37 °C without light. The gates were set as in the multicolor assay to exclude doublets and make sure that only single cells of sperm population were measured. The mean fluorescence of HE in cells with M_pos_ is defined as “ROS synthesis” and the mean fluorescence of Bodipy in cells with M_pos_ is defined as “Lipid peroxidation” (LPO).

### 2.7. Seahorse Metabolic Assay

The Seahorse XFp extracellular flux analyzer (Agilent Technologies, Inc., Santa Clara, CA, USA) was used to measure mitochondrial activity via oxygen consumption rate (OCR) and extracellular acidification rate (ECAR) in real time. For each ejaculate, basal values were determined, showing the regular metabolism of stallion sperm without manipulation. The MitoStress Test was additionally performed to examine mitochondrial metabolism in more detail. This test involves three injections conducted automatically by the Seahorse Analyzer. Initially, oligomycin, which inhibits ATP-synthase, was injected after basal value measurement. Subsequently, the respiratory chain was uncoupled by injection of FCCP (carbonyl cyanide-p-trifluoromethoxy phenyl-hydrazone), which induces maximal mitochondrial respiration and increases the OCR to the maximum. The final injection contained antimycin A and rotenone, which block complexes III and I of the respiratory chain, respectively, resulting in complete inhibition of the respiratory chain, which is reflected in a decreased OCR.

Eighteen hours prior to the measurement, the Seahorse XFp Analyzer was turned on to reach the desired temperature of 37 °C. The sensor cartridge was hydrated with sterile water and placed in an incubator at 37 °C. The 8 wells of the cell culture miniplate were coated with 15 µL of concanavalin A (0.5 mg/mL) and left to dry at room temperature until starting the experiment (Supplementary Figure S1).

One hour prior to semen seeding, the cartridge calibration was prepared by replacing the sterile water with XF Calibrant and incubation at 37 °C for 45–60 min.

To perform the MitoStress Test, reagents were prepared and placed in the ports of the sensor cartridge (Supplementary Figure S2). For the first injection, 20 µL of oligomycin working solution was pipetted into port A, resulting in a final concentration of 2 µM oligomycin per well. For the FCCP injection, 22 µL was added to port B for a final concentration of 4 µM FCCP per well. Port C was loaded with 25 µL of antimycin A and rotenone working solution, with a final concentration of 1 µM of each per well. The ports of the wells without reagent were filled with the same volumes of Tyrode’s solution as negative control.

Two wells were used for one ejaculate to measure basal OCR and ECAR without injections of mitochondrial reagents, and the other well was used to perform the MitoStress Test (Supplementary Figure S1). The coated culture plate was loaded from position B-G with 20 µL of raw semen to achieve a concentration of 2–6 × 10^6^ sperm per well. Position H was used as reference. The wells were then filled with 160 µL of Tyrode’s solution to a total volume of 180 µL/well. Position A was only filled with 180 µL of Tyrode’s solution for background measurements. Each of the outer chambers were filled with 100 µL of PBS, and the culture plate was centrifuged at 300× *g* for 1 min at 23 °C, then rotated 180° and centrifuged again for 1 min. Forty µm nylon meshes (saturated in Tyrode’s solution) were inserted into each well to keep the sperm at the bottom. Once the calibration of the Seahorse was completed, the culture plate was loaded into the device. After 2 min, the first measurement was carried out 15 min after thawing of the sperm samples.

The configuration of the assay was performed with the Seahorse Wave Desktop software version 2.6 (Agilent Technologies, Inc., Santa Clara, CA, USA). Cycles consisted of a 2 min waiting period and a 3 min measurement period without mixing. To determine baseline values, four cycles were initially performed, followed by three cycles per injection. However, basal measurements of OCR and ECAR were also carried out for a total of 120 min in parallel to the other assays.

Afterwards the sperm was resuspended and the concentration in each well was then determined using CASA to calculate the actual cell count with the known volume of 247 µL per well at the end of the assay. The values for OCR and ECAR were then normalized to 1 million cells [25,31].

### 2.8. Study Design

**Figure 1:**
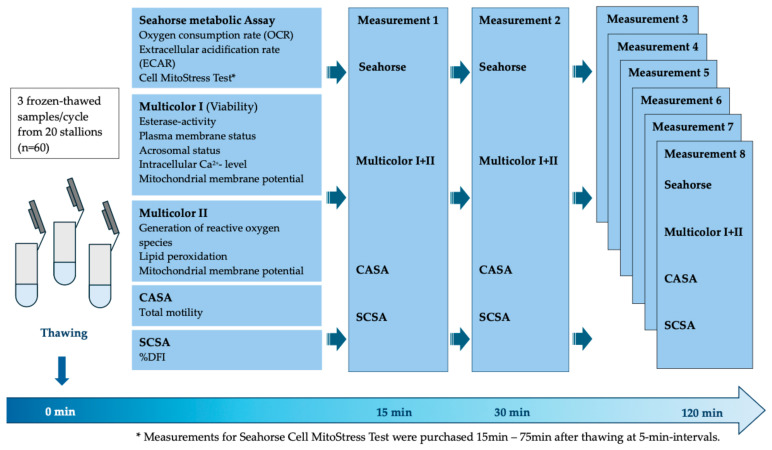
Study design of characterization of cryopreserved stallion sperm using the Seahorse Analyzer, CASA, and multiparametric flow cytometry. Three cryopreserved semen samples from each of the 20 Warmblood stallions (Holsteiner Verband) were analyzed using five different assays in parallel over a period of 120 min post-thaw by measurements every 15 min at a constant temperature of 37 °C. The sperm metabolism was examined using the Seahorse Analyzer by measuring basal OCR and ECAR without manipulation and, in parallel, the MitoStress Test was performed to reveal more details about metabolism in stallion sperm. The total motility of the sperm was assessed by CASA. Furthermore, three flow cytometric assays were carried out to evaluate viability, ROS synthesis, and LPO. The SCSA™ test was performed to determine the %DFI in each sperm sample.

The quality of three cryopreserved semen samples from each of 20 Warmblood stallions (Holsteiner Verband) was characterized using five different assays in parallel over a period of 120 min by measurements every 15 min at a constant temperature of 37 °C (Figure 1). The sperm metabolism was examined using the Seahorse Analyzer by measuring basal OCR and ECAR without manipulation and, in parallel, the MitoStress Test was performed to reveal more details about metabolism in stallion sperm. The total motility of the sperm was assessed by CASA. Furthermore, three flow cytometric assays were carried out to evaluate viability, ROS synthesis, and LPO. The SCSA™ test was performed to determine the %DFI in each sperm sample.

### 2.9. Statistical Analysis

**Figure 2:**
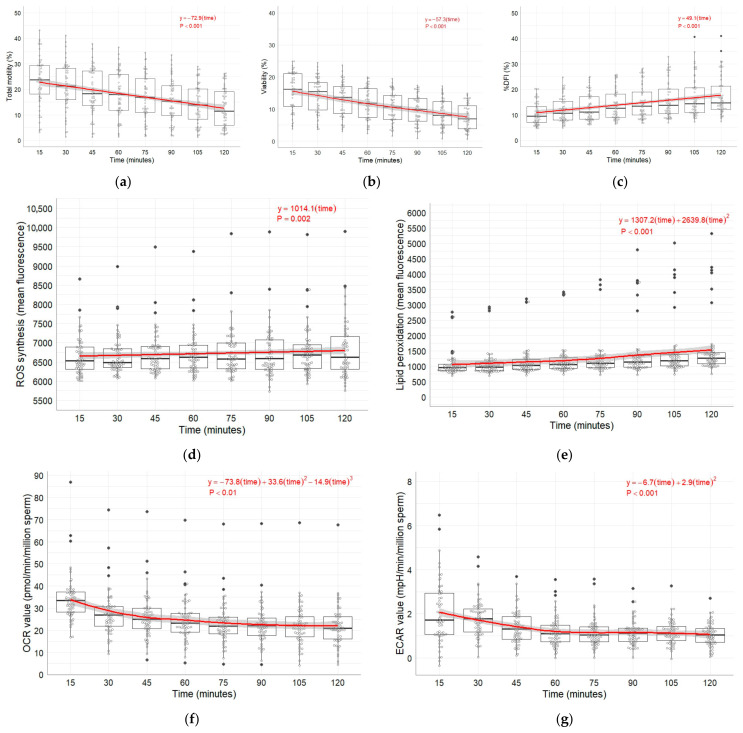
Changes in total motility (**a**), viability (**b**), DNA damage ((**c**); %DFI), ROS synthesis (**d**), lipid peroxidation ((**e**); LPO), oxygen consumption rate ((**f**); OCR), and extracellular acidification rate ((**g**); ECAR). Sixty frozen–thawed ejaculates collected from 20 stallions (three ejaculates per stallion). Values are shown as boxplots, the red line in the boxplots represents a locally estimated scatterplot smoothing (LOESS) curve, which provides a smoothed representation of the trend over time. The displayed equation corresponds to the fitted LOESS model.

**Figure 3:**
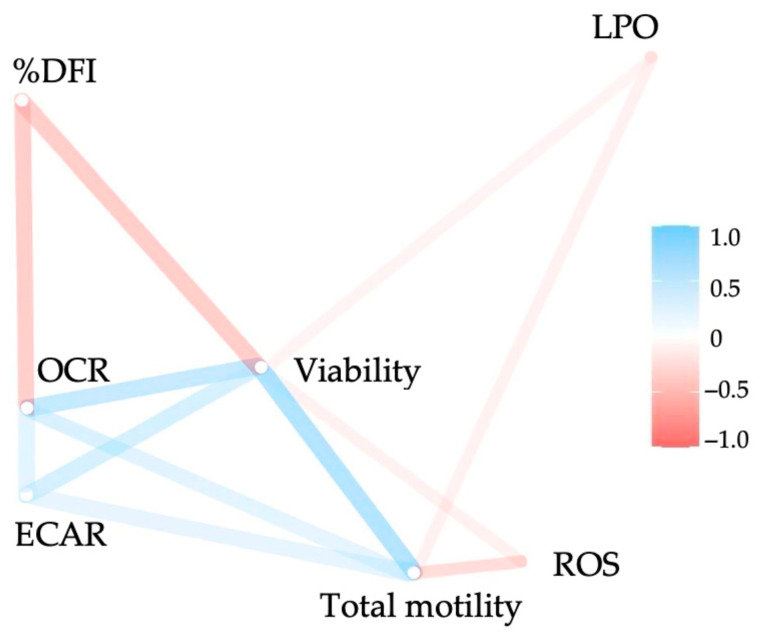
Network plot for pairwise correlations between sperm quality traits (assessed with computer-assisted sperm analysis and flow cytometry) and the values of OCR and ECAR evaluated at 15 min intervals between 15 and 120 min after thawing. Sixty frozen–thawed ejaculates collected from 20 stallions (three ejaculates per stallion) were examined between 15 and 120 min after thawing. Variables that are more highly correlated appear closer together and are joined by stronger (more dens color) paths. Paths are also colored by their sign (blue for positive and red for negative correlations). The proximity of the points was determined using multidimensional clustering.

Statistical analyses were performed using the R language and environment for statistical computing (version 4.2.2) [38]. Descriptive statistics (median, interquartile range, mean ± SD) were calculated for all sperm quality traits determined with CASA, flow cytometry and Seahorse Analyzer. Differences in temporal trends were evaluated using locally estimated scatterplot smoothing (LOESS) models. The corresponding fitted equations are presented in Figure 2. Coefficients of variation (CV) were determined to assess between- and within-stallion variabilities. Pairwise correlations between sperm parameters within each examination time point were analyzed using Spearman’s rank correlation coefficients (r_s_) at 0.05 significance level. A correlation network plot was created to visualize these correlations across all different time points (Figure 3).

Time series clustering was performed to identify groups of stallions exhibiting similar temporal patterns of OCR and ECAR values during the MitoStress Test. The input dataset included the variables stallion (individual identifier), time (measurement time points), and the normalized values for OCR and ECAR (per million of cells examined in each sample). Prior to clustering, time series were standardized using z-score normalization. Partitioning clustering was conducted using the k-means algorithm with dynamic time warping (DTW) as the distance metric (dtwclust package). The optimal number of clusters (k = 2) was selected based on interpretability and cluster stability. The following R packages were used: dtwclust [39] (for time series clustering), dplyr [40] (for data manipulation), and reshape [41] (for data reshaping). Stallions were then assigned to DTW-based clusters according to their OCR response profiles.

To investigate the effect of measurement time, stallion age, and DTW-based clustering following the MitoStress (MitoStress clusters) on sperm quality traits and Seahorse parameters, linear mixed-effects models were fitted using the lme() function from the nlme [42] package. The model included a third-degree polynomial of time, stallion age in months (age.mo), and the MitoStress clusters as fixed effects; the individual identifier of each stallion as a random effect to account for repeated measures within individuals; a first-order correlation structure to account for temporal autocorrelation. Model parameters (estimates of b coefficients for the fixed effects with 95% confidence intervals) were considered significant for *p* values < 0.05.

The temporal dynamics of mitochondrial activity and their relationship to DTW-based clusters were further assessed through two separate two-way analyses of variance (ANOVA) for the normalized values of OCR and ECAR (dependent variables), respectively. The dependent variables were modeled as a function of two categorical independent variables: time interval and MitoStress clusters. Measurement time points during the MitoStress Test were grouped into six intervals: 15 min, 20–30 min, 35–45 min, 50–60 min, 65–75 min, and 90+ min. The interaction between time interval and MitoStress clusters was included in both ANOVAs to evaluate whether the temporal progression of OCR or ECAR differed between clusters. Following significant ANOVA results, Tukey’s Honest Significant Difference (HSD) post hoc tests were conducted to identify pairwise differences among time groups and between DTW-based clusters.

## 3. Results

The ejaculates used for the study showed no macroscopic aberrations in color, odor, admixtures, and consistency and fulfilled the qualitative standard requirements in motility to be cryopreserved [43].

### 3.1. Basal Measurements

#### 3.1.1. Descriptive Statistics

The descriptive statistic measures (n, median, interquartile range (IQR), arithmetic mean ± SD) for the OCR- and ECAR values of the Seahorse basal measurements as well as CASA and flow cytometric sperm traits assessed at time points 15, 30, 45, 60, 75, 90, 105, 120 min after thawing are presented in Supplementary Table S1. Total motility and viability decreased linearly during the examination period (Figure 2a,b), while in contrast, %DFI and ROS synthesis rose in a linear manner at the same time (Figure 2c,d). Lipid peroxidation increased (Figure 2e) and ECAR values declined over time, followed by a quadratic form (Figure 2g). Only the time curve of the OCR values followed a decreasing cubic shape (Figure 2f).

The mean coefficient of variation (CV) between different stallions was higher in all parameters (mean ± SD: 36.21 ± 13.4%) compared to within-stallion CV (mean ± SD: 24.47 ± 13.45%). The inter-stallion CV of ROS synthesis was lower compared to other evaluated parameters (Table 1). High intra-stallion variabilities were found in all sperm parameters except for ROS synthesis (Table 1).

#### 3.1.2. Correlations

The results of the pairwise correlation analysis of sperm parameters are presented in Supplementary Table S2. Figure 3 visualizes these correlations graphically in a network plot. The strongest positive correlations were found between OCR and ECAR, and between total motility, viability, and Seahorse parameters (OCR and ECAR). The most pronounced negative correlations occurred between %DFI and viability, as well as between %DFI and OCR. Only weak or no correlations were observed between oxidative stress markers (ROS and LPO) and the other sperm parameters.

Total motility was positively related to viability (r_s_ = 0.49, *p* < 0.01; Supplementary Table S2) and OCR- (r_s_ = 0.43, *p* < 0.01; Supplementary Table S2) and ECAR values (r_s_ = 0.56, *p* < 0.01; Supplementary Table S2). There were no significant relationships between total motility and %DFI and LPO. Viability was positively related to OCR (r_s_ = 0.52, *p* < 0.01; Supplementary Table S2) and ECAR (r_s_ = 0.31, *p* = 0.02; Supplementary Table S2). While viability and total motility showed no correlation with ROS synthesis at the beginning of the measurements, an increasingly negative correlation was observed over the 2 h incubation period (viability after 2 h: r_s_ = −0.52, *p* < 0.01; total motility after 2 h: r_s_ = −0.44, *p* < 0.01; Supplementary Table S2). Viability was not significantly associated with LPO. The %DFI values were negatively related to OCR (r_s_ = −0.33, *p* < 0.01; Supplementary Table S2) and viability (r_s_ = −0.39, *p* < 0.01; Supplementary Table S2). There was no significant relationship between ROS synthesis and lipid peroxidation, OCR- and ECAR values, respectively. OCR- and ECAR values were positively related to each other (r_s_ = 0.69, *p* < 0.01; Supplementary Table S2).

### 3.2. MitoStress Test

**Figure 4:**
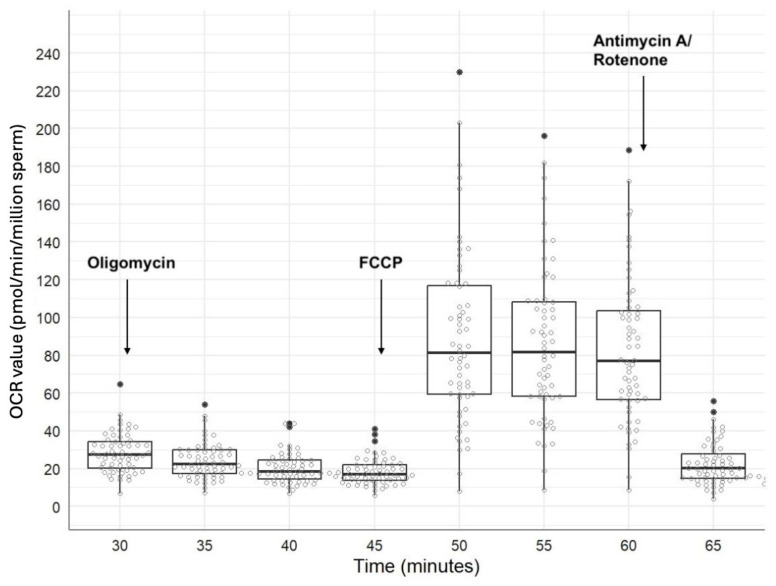
Boxplots for the OCR values at 5 min intervals between 30- and 65 min post-thaw after challenging sperm with MitoStress agents. Data are from sixty frozen–thawed ejaculates collected from 20 stallions (three ejaculates per stallion).

**Figure 5:**
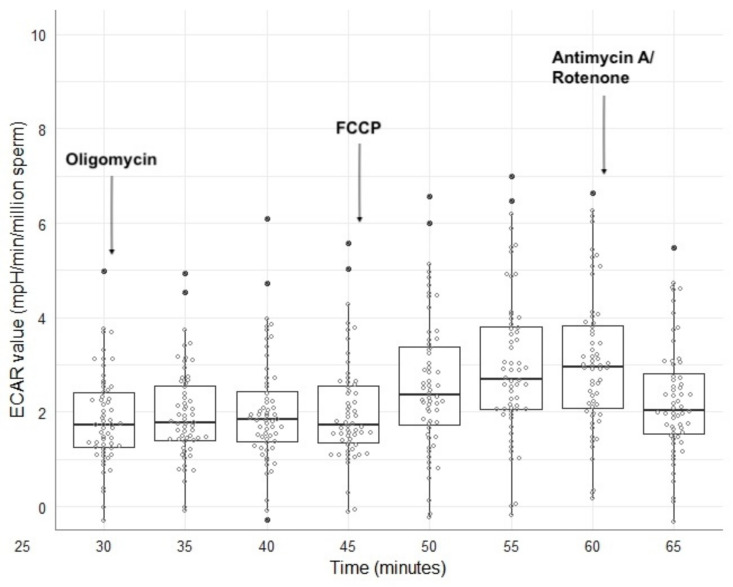
Boxplots for the ECAR values at 5 min intervals between 30- and 65 min post-thaw after challenging sperm with MitoStress agents. Data are from sixty frozen–thawed ejaculates collected from 20 stallions (three ejaculates per stallion).

**Figure 6:**
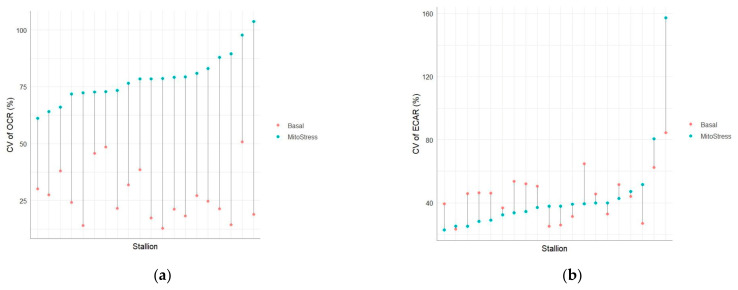
Coefficients of variation (CV, within-stallion variability) for (**a**) OCR- and (**b**) ECAR values assessed in 60 frozen–thawed ejaculates collected from 20 stallions (three ejaculates per stallion) after basal Seahorse measurements and challenging with MitoStress agents.

The descriptive statistics for the OCR- and ECAR values recorded after applying the MitoStress Test at 5 min intervals between 15- and 75 min post-thaw are shown in Supplementary Table S3. MitoStress Test results are illustrated in Figure 4 and Figure 5 for OCR- and ECAR values, respectively. Once the basal values were recorded 30 min after thawing, the first injection of oligomycin was performed, resulting in a decrease in OCR values (Figure 4). There was no reaction of ECAR values after the oligomycin injection (Figure 5). All stallions showed an increase in OCR- and ECAR values after FCCP injection 45 min after thawing (Figure 4 and Figure 5). The antimycin A/rotenone injection 60 min post thaw led to a decrease in OCR values below basal values (Supplementary Table S3). There was also a decrease in ECAR values (Figure 4 and Figure 5). Across all stallions, the OCR values showed a higher between-stallion variability in MitoStress measurements than in basal measurements (Table 2). On the other hand, the CVs for the values of ECAR were similar between the basal and MitoStress measurements (Table 2). For all stallions, the within-stallion variability of OCR was higher after MitoStress than OCR during basal measurements (Figure 6a). There were no differences in CVs for ECAR values between basal measurements and after MitoStress (Figure 6b).

#### Time Series Clustering and Cluster-Specific Differences in Mitochondrial Response Patterns

**Figure 7:**
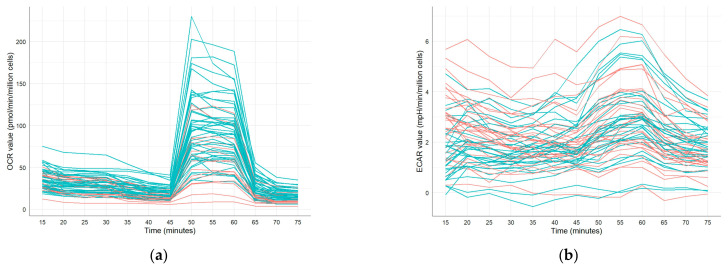
Time series of (**a**) OCR- and (**b**) ECAR values of frozen–thawed equine sperm after MitoStress challenging for two different clusters of stallions (red: Cluster 1, blue: Cluster 2). Clusters were defined by performing a dynamic time warping-based partitioning. Sixty frozen–thawed ejaculates collected from 20 stallions (three ejaculates per stallion) were examined.

In Figure 7, the temporal sequences of OCR- and ECAR values (after MitoStress challenging) are shown for two different clusters of stallions. The two Clusters 1 and 2 defined for OCR values had a size of 5 and 15 stallions with an intra-cluster distance of 3.92 and 1.87, respectively. As shown in Figure 7a, five stallions with OCR values < 50 pmol/min/million cells at 50–60 min (after FCCP injection) were assigned to Cluster 1, while all males with OCR values > 125 pmol/min/million cells at this time point belong to Cluster 2. Using the time series of ECAR values to detect clusters of stallions with similar ECAR patterns over time, the two identified Clusters 1 and 2 were not clearly distinct (with sizes of 10 and 10 stallions, and intra-cluster distance of 6.56 and 6.03, respectively; Figure 7b). According to the results of the analysis of variance (ANOVA; Supplementary Table S4), different responses to the MitoStress injections were observed between stallions in Cluster 1 and Cluster 2. Spermatozoa in Cluster 1 did not show a significant reaction to oligomycin, whereas a significant decrease in OCR values was detected in Cluster 2 (*p* = 0.04). ECAR values remained unchanged in both clusters following oligomycin injection. In response to FCCP, both clusters exhibited significant increases in OCR- and ECAR values (*p* < 0.01). The maximal respiration reached in Cluster 2 was significantly higher than in Cluster 1 (*p* < 0.01). Following the injection of antimycin A and rotenone, both clusters showed a significant decrease in OCR and ECAR (*p* < 0.01).

### 3.3. Relationships Between Age of Stallions, Oxygen Consumption Rate, Extracellular Acidification Rate, Flow Cytometric Parameters, and Total Motility of Sperm

To investigate relationships between stallion age and the group of MitoStress response on sperm quality traits and the values of OCR and ECAR recorded during the Seahorse basal measurements, we used linear mixed-effects models. The output of each model describing the effect of the above-mentioned fixed effects on the sperm quality traits are presented in Supplementary Table S5. Total motility and viability deteriorated with progressing age of the stallion (b < 0, *p* < 0.05), while %DFI, ROS, and LPO were not significantly affected by increasing animal age (*p* > 0.05 for the b estimates of these fixed effects; Supplementary Table S5). The effect of age was also proven non-significant for values of OCR and ECAR (Supplementary Table S5). It appears that differences between stallions occurred not only in the response pattern of the OCR values after the MitoStress challenging, but also during the basal OCR measurements, with animals of Cluster 2 showing higher OCR records compared to these of Cluster 1 (b = 6.49, *p* = 0.04; Supplementary Table S5). The same trend was also observed for the ECAR values but failed to reach levels of statistical significance (b = 0.48, *p* = 0.06; Supplementary Table S5).

## 4. Discussion

The comparative analysis of established sperm quality parameters in this study revealed a linear decline in viability and motility over a 120 min post-thaw period. While this trend is well-documented in the literature [8,9], the assessment at short intervals post-thaw, as applied here, provides novel insight into the temporal resolution of this decline. Comparability of viability values between studies is challenging, as the definition of sperm viability is not standardized and there is substantial methodological heterogeneity [44]. A similar issue of comparability exists for motility assessment, as comparisons between CASA studies on stallion semen are limited due to variability in devices, software settings, sample preparation, and analysis conditions, making cross-study interpretation difficult [45,46]. As expected, %DFI values increased linearly during the post-thaw observation period. A similar rise in DNA fragmentation has been previously demonstrated [47]. The %DFI values reported in the present study (%DFI mean ± SD: 10.43 ± 4.35%; 15 min after thawing, Supplementary Table S1) are comparable to those found in other studies on cryopreserved stallion sperm [48,49], although the substantial inter-individual variability typical for equine semen should be considered [50].

Consistent with the general consensus [16], ROS synthesis and LPO also increased over time after thawing. Under physiological conditions, however, healthy sperm maintain a delicate redox balance through enzymatic antioxidant systems that neutralize excess ROS and prevent oxidative damage [51,52]. When redox regulation is disrupted, caused by cryoinjuries, increased reactive oxygen species initiate lipid peroxidation, compromising membrane integrity and mitochondrial function. This cascade reduces ATP availability and thereby limits sperm viability and motility [53,54], providing a plausible link between oxidative stress, mitochondrial function, and the observed changes in conventional sperm quality parameters.

In our experiment, basal OCR and ECAR displayed a cubic trajectory, showing a steep initial decline, whereas the other examined parameters followed a linear trend across the measurement period (Figure 2). As the Seahorse assay specifically measures the metabolic activity of viable spermatozoa, the observed steady decline in viability during incubation, in contrast to the dynamic changes in OCR and ECAR, highlights the high sensitivity of this approach in detecting subtle metabolic alterations at an earlier stage than conventional methods such as flow cytometry or CASA. A similar OCR pattern was observed in bovine sperm of different breeds after selection of sperm using the swim-up method [27]. Although the measurement period in that study was only 22 min, the OCR values of the upper fraction, which exhibited higher motility, revealed a curve progression comparable to that observed in stallion sperm in our study. In contrast, both the lower fraction and the measurement prior to the swim-up method displayed lower and stable OCR values over time. Notably, ECAR values were also markedly increased in the upper fraction, indicating a relative metabolic shift towards glycolysis. These findings are consistent with previous reports highlighting metabolic differences between bovine and stallion sperm [20,55].

During basal measurements, the recorded OCR values in our study were generally lower than those reported in a study using fresh stallion semen [33], most likely due to the metabolic impairments caused by cryopreservation. To date, there exists no direct comparison of OCR- and ECAR values between fresh and frozen–thawed equine sperm, whereas such data are available for bovine sperm [31]. The findings of this study indicate that frozen–thawed semen of bulls exhibits a higher basal respiration compared to fresh semen. This contrary finding may be related to the greater metabolic flexibility of bovine sperm in terms of energy substrate utilization [56]. OCR values for human spermatozoa have been shown to be lower than those obtained in the present study [25], which likely reflects the preferred use of glycolysis as the main metabolic pathway in human sperm [57]. Previous studies have demonstrated that cryopreserved spermatozoa show premature capacitation-like changes and early signs of hyperactivation due to membrane destabilization, increased intracellular calcium, and elevated ROS production [58,59,60,61]. These physiological changes do not only compromise membrane integrity but also induce excessive flagellar activity, which significantly increases ATP consumption. As a result, the available mitochondrial energy reserves are rapidly depleted, potentially contributing to the reduced motility values observed in extended post-thaw incubation. While we also observed a decrease in ECAR during the two-hour basal measurements in equine sperm, Magdanz et al. [27] reported an increase in ECAR in bull sperm. This difference may be explained by the greater dependence of bull sperm on glycolysis [62], whereas glycolysis plays only a minor role in stallion sperm metabolism.

When interpreting the Seahorse-derived OCR in stallion spermatozoa, the potential impact of dead or apoptotic sperm cells within the sample should be noted. During cryopreservation and prolonged incubation, a proportion of spermatozoa inevitably undergoes membrane damage and apoptosis-like changes, characterized by mitochondrial depolarization, caspase activation, and increased reactive oxygen species production [60,63]. These non-viable cells may not only fail to contribute to mitochondrial respiration themselves but can also release factors such as apoptotic bodies and ROS into the surrounding medium [11,51]. Such by-products can impair the metabolic activity of neighboring viable sperm by promoting oxidative stress, lipid peroxidation, and further mitochondrial dysfunction, effectively acting as ROS amplifiers that compromise the efficiency of otherwise functional spermatozoa [52,64,65,66]. Consequently, the measured OCR may not exclusively reflect the genuine bioenergetic capacity of the intact sperm population but could be partly depressed by the presence of apoptotic cells.

A positive correlation between mitochondrial membrane potential, measured by flow cytometry, and sperm motility has been demonstrated, underlining the central role of mitochondrial function for sperm vitality and flagellar motion [7,60,66]. The correlation patterns observed in this study partially align with these findings, as we also identified positive relationships between OCR, ECAR, and conventional parameters such as motility and viability, highlighting that metabolism is the basis for sperm function. This is further supported by previous studies demonstrating positive relationships between OCR, motility, and viability in human and equine semen [22,67]. Interestingly, ECAR showed strong positive correlations with sperm motility (Supplementary Table S2). This finding suggests that ATP required for motility is predominantly generated through OXPHOS but can also be supplemented by glycolysis [68], particularly under cryo-induced stress conditions. Furthermore, when mitochondrial ATP production is impaired by cryo-induced damage, glycolysis may be upregulated as a compensatory mechanism to maintain cellular energy homeostasis [69]. In addition, capacitation-like changes induced by cryopreservation increase the energetic demand of sperm and promote a metabolic shift towards glycolysis [70]. The Seahorse technology is a promising approach to investigate metabolic changes associated with capacitation in stallion sperm, as it has already been applied in other species [24,71]. By using controlled injections during the assay, capacitation can be experimentally induced, allowing real-time monitoring of the metabolic responses.

The negative correlation between %DFI and OCR found here confirms that higher mitochondrial activity is generally associated with lower DNA fragmentation. This is in line with previous work indicating that mitochondrial dysfunction may promote oxidative damage to sperm DNA [60]. Interestingly, while viability and motility showed no clear correlation with ROS synthesis at the beginning of incubation, a progressively negative correlation emerged over time. This indicates that increasing ROS production during prolonged incubation gradually impairs membrane integrity and motility, as already described [52,64]. It should be noted, however, that ROS and LPO values cannot be directly compared with conventional sperm quality parameters such as motility or viability, as they are based on different methodological principles. While ROS and LPO represent the extent of oxidative activity within the viable sperm, motility and viability are expressed as percentages of the total sperm population. Nevertheless, the dual role of ROS is well established: moderate levels support sperm capacitation and motility by activating signaling pathways, whereas excessive ROS generation shifts this balance towards lipid peroxidation and mitochondrial dysfunction, ultimately compromising sperm function [51,72].

Contrary to our expectations, however, we did not find any correlations between LPO and other sperm parameters, not even between LPO and ROS, despite this association being well-documented in different species [73,74,75,76]. The lack of correlation between LPO and ROS observed in this study may be explained by a temporal delay between the generation of ROS and the initiation of LPO. ROS are produced immediately after thawing, whereas lipid peroxidation develops more gradually as a consequence of ROS synthesis [2]. As a result, ROS levels may have already declined at the time LPO becomes measurable, masking a potential relationship between both parameters. In addition, inter-individual variability among stallions may contribute to the heterogeneity of oxidative stress responses. An innovative approach would be to incorporate lipidomic profiling to identify stallion-specific features in membrane composition and to determine lipid classes that are particularly susceptible to peroxidation [77]. The integration of these insights with Seahorse technology would facilitate the controlled induction of ROS and LPO through specific injections, allowing simultaneous assessment of potentially protective effects of antioxidants or extender components.

We found substantial inter-stallion variabilities for most measured parameters, except for ROS (Table 1). This finding aligns with the well-established understanding that stallion semen is characterized by considerable biological variation [50,63]. In contrast, ROS levels showed only little variation between individuals in this study, indicating that this parameter is not well suited to differentiate among stallions. Compared to Battut et al. [9], we observed higher inter-stallion CVs for viability and motility (Table 1), but viability was assessed using a different technique in each study. In contrast, the CV for %DFI was considerably lower in our study (Table 1). While no published CVs are currently available for OCR- and ECAR values in sperm, the variability observed in our data is within a similar range as that of established parameters such as motility and viability. This suggests that the Seahorse Analyzer is well suited to detect inter-individual differences in stallion sperm metabolism. In line with other studies, we observed higher variabilities between stallions (CV: 36.21%) than within stallions (CV: 24.74%) across all measured sperm quality traits [9,78,79].

Among the conventional parameters, we observed high intra-individual variabilities for total motility (CV: 32.39%, Table 1), viability (CV: 31.75%, Table 1), and %DFI (CV: 19.71%, Table 1). This is in contrast with other findings where DNA fragmentation showed higher intra-individual variability than motility and viability parameters in equine sperm [9]. ROS showed the lowest coefficient of variation among all measured parameters. One possible explanation for the limited variability of ROS and LPO is the difference in measurement principles compared to conventional parameters. While viability, motility, and %DFI are expressed as proportions of the total sperm population, ROS and LPO represent fluorescence intensity within the viable fraction, which makes a direct comparison of variability across methods difficult. Interestingly, the Seahorse assay, which likewise quantifies the metabolic activity of viable spermatozoa, yielded CVs in a range similar to those of conventional parameters such as viability, motility, and %DFI. Another potential explanation is a relatively high basal level of oxidative stress in the samples, likely induced by cryopreservation, which may have masked individual differences. This hypothesis is further supported by the fact that stallions, due to their high OXPHOS activity in spermatozoa, generally exhibit elevated ROS levels [14]. In addition, cryopreservation itself has been shown to increase both ROS production and lipid peroxidation [80], potentially contributing to a uniformly elevated oxidative status across samples. A direct comparison of absolute ROS and LPO values with other studies is not possible, as most published data report only relative changes rather than absolute values [81,82,83]. Unfortunately, comparable data investigating intra-individual variation in ROS and LPO in stallions are lacking. Moreover, the comparison of absolute values across studies is of limited validity, as these measurements are strongly influenced by differences in flow cytometric equipment and analytical settings.

The within-stallion CV for OCR (CV: 27.34%, Table 1) was in a similar range as CVs for motility and viability, while the CV for ECAR was even higher (CV: 44.46%, Table 1). The predominantly high values of intra-individual variability may have multiple causes. One possible explanation is an ejaculate-specific effect inherent to the samples themselves. Battut et al. [9] previously pointed out that at least six ejaculates per stallion are needed to make a reliable statement of stallion’s semen quality. Another important factor could be seminal plasma, which has been shown to influence the freezability of ejaculates due to varying antioxidant capacities [84]. As a result, it also affects sperm quality directly [85]. Stallion-specific differences in the composition of seminal plasma have already been demonstrated, suggesting that an ejaculate-specific effect may also exist [86]. Another potential source of variation could be the semen handling process itself, from collection, freezing, storage through the execution of the assays.

After application of the MitoStress Test, the variability of OCR increases (CV: 78.42%, Table 2) in contrast to ECAR (CV: 44.04%, Table 2), showing stable variability of ECAR values across MitoStress challenging. These findings suggest that the MitoStress Test can uncover additional ejaculate-specific differences via OCR, whereas ECAR appears less informative in this regard, which likely reflects the dominant role of oxidative phosphorylation in stallion sperm compared to glycolysis [14,20].

In our study, stallion sperm showed individually variable responses to the MitoStress reagent, but each ejaculate exhibited a measurable reaction in OCR (Figure 4). In contrast to our data, a previous study using fresh stallion semen reported higher basal OCR values and more pronounced decrease in OCR following oligomycin injection [33]. Interestingly, in that study, maximal respiration after FCCP injection did not exceed basal respiration, which differs from our observations in cryopreserved semen (Figure 4). A study on fresh boar semen showed clear responses to MitoStress Test [32], comparable to those described for human sperm [25]. Basal OCR values of fresh boar semen were also higher than those observed in our frozen–thawed samples. We observed that stallion spermatozoa exhibited no increase in ECAR following oligomycin injection but respond with an ECAR rise only after FCCP (Figure 5). In human sperm, however, oligomycin alone is sufficient to trigger a glycolytic shift [25], indicating a higher preference for glycolysis [87]. Algieri et al. [30] reported that cryopreserved bull spermatozoa failed to respond to FCCP, with no increase in OCR, which they attributed to mitochondrial damage caused by cryopreservation. Moraes et al. [31] compared fresh and frozen–thawed bull semen and found that maximal respiration was higher in frozen–thawed sperm than in fresh samples. A similar effect is observed when comparing the findings of our study on cryopreserved equine semen with those reported for fresh stallion semen [33].

The clustering observed in the MitoStress Test results clearly indicates that the stallions can be grouped based on their OCR into two distinct clusters with low and high OCR at 50–60 min post-thaw (Figure 7). This time point corresponds to the maximum respiration phase following FCCP injection, reflecting the cells’ maximum mitochondrial capacity. Cluster-specific differences were not only observed following FCCP injection. Stallions in Cluster 1 showed no significant response to oligomycin, whereas those in Cluster 2 exhibited a significant change in OCR (*p* = 0.04, Supplementary Table S4). Both clusters responded to FCCP with an increase in OCR (*p* < 0.01, Supplementary Table S4), but the increase was more pronounced in Cluster 2 compared to Cluster 1 (*p* < 0.01, Supplementary Table S4). This clustering effect was not observed with any of the other assays applied in this study, including flow cytometry and CASA parameters. This suggests that Seahorse-based bioenergetic profiling may capture subtle functional characteristics of mitochondrial performance that remain undetected by conventional semen evaluation methods. These findings are consistent with previous studies showing that mitochondrial function is closely related to sperm cryotolerance [88] and highlight the added diagnostic value of metabolic stress testing in the context of stallion semen preservation.

Age-related effects on stallion sperm quality are well-documented in the literature, particularly with regard to reduced freezability, declining motility, and mitochondrial efficiency [50,89,90]. However, more recent studies have emphasized that substantial inter-individual variations among stallions may mask the impact of age and seasonality, while also interacting with additional factors such as stallion management [91,92]. This could explain why in our study no age effect could be detected for any parameter except viability and total motility. These findings highlight that age can influence specific aspects of sperm function, but its effects are not necessarily uniform across all quality parameters.

The Seahorse system is still expensive and requires specific technical instruction, which currently limits its use for routine practice in the field. Furthermore, only one study on stallion sperm has been published to date [33], and the method needs to be further established and standardized for stallion reproduction research. However, stud farms have the option to collaborate with laboratories that are equipped with this technology for specific research requests. The Seahorse Analyzer could be a valuable tool for various research purposes, including comparing different extenders and cryopreservation protocols. It can also be used to investigate bioenergetic aspects of sperm freezability. In addition, combining Seahorse metabolic assay with metabolomics approaches offers promising opportunities to deepen the understanding of sperm metabolism and to improve cryopreservation strategies. Beyond metabolomics, lipidomics analyses would represent another highly relevant complementary approach, as previously discussed. Integrating Seahorse-based metabolic profiling with lipidomic data could provide novel insights into how membrane lipid composition affects sperm bioenergetics and cryotolerance. Potential extensions of such studies might also include dietary interventions, such as the supplementation of stallions with specific feed additives designed to alter the lipid composition of sperm membranes [93]. The resulting effects on post-thaw metabolic activity and on the freezability of the modified lipid profile could then be evaluated using the Seahorse Analyzer.

It is crucial that future studies integrate fertility data, as establishing associations between metabolic parameters and fertilization success will be essential for translating these findings into practical breeding use.

## 5. Conclusions

In summary, the present study demonstrates the strong diagnostic potential of functional mitochondrial profiling using Seahorse XFp technology. By capturing real-time metabolic activity, this method provides additional insights that complement conventional sperm quality assays such as CASA and flow cytometry. Our results underline the importance of a multiparametric assessment that combines structural, functional, and metabolic data to achieve a more comprehensive understanding of stallion sperm quality. The marked intra- and inter-stallion variabilities observed in this study confirm the high individual diversity typical for stallions. In the future, Seahorse technology will provide valuable opportunities to further investigate the relationship between metabolism and fertility and to improve cryopreservation strategies in stallion sperm.

## Figures and Tables

**Table 1:** The coefficient of variation (CV) within and between stallions for the values of total motility (%), viability (%), DNA damage (%DFI, %), ROS synthesis (mean fluorescence), lipid peroxidation (mean fluorescence), OCR (pmol/min/million sperm), and ECAR (mpH/min/million sperm). Three frozen–thawed semen samples from each of the 20 stallions were examined between 15- and 120 min after thawing.

Sperm Parameter	Within-Stallion CV, % Mean ± SD (Range)	Between-Stallion CV, %
Total motility	32.39 ± 13.81 (13.43–59.68)	42.64
Viability	31.75 ± 15.46 (15.17–69.46)	39.08
%DFI	19.71 ± 5.58 (11.62–33.77)	40.8
ROS	3.98 ± 2.21 (1.78–9.54)	7.85
LPO	13.53 ± 15.64 (4.88–77.33)	45.68
OCR	27.34 ± 11.55 (12.82–50.75)	31.62
ECAR	44.46 ± 15.34 (23.26–84.47)	45.77

**Table 2:** The coefficient of variation (CV, %) for the values of OCR and ECAR assessed in 60 frozen–thawed ejaculates collected from 20 stallions (three ejaculates per stallion), after basal Seahorse measurements and challenging with MitoStress agents.

Characteristic	Overall	Basal	MitoStress
	N = 1260 ^1^	N = 480 ^1^	N = 780 ^1^
CV (%) of OCR	58.96 ± 26.99	27.34 ± 11.27	78.42 ± 10.22
CV (%) of ECAR	44.19 ± 24.34	44.44 ± 14.92	44.04 ± 28.64

^1^ Mean ± SD.

## Data Availability

The original contributions presented in this study are included in the article/Supplementary Materials. Further inquiries can be directed to the corresponding author(s).

## Supplementary Materials

The following supporting information can be downloaded at: https://www.mdpi.com/article/10.3390/vetsci12121109/s1. Figure S1: Schematic overview of the Seahorse culture plate setup and semen seeding procedure prior to Seahorse assay; Figure S2: Preparations of injections to perform the MitoStress Test; Table S1: Descriptive statistic measures for sperm quality traits of basal measurements; Table S2: Spearman’s correlation coefficients for the pairwise correlations between sperm quality traits; Table S3: Descriptive statistic measures for the sperm quality traits assessed with the Seahorse platform after challenging sperm with MitoStress Test; Table S4: Two-way analysis of variance (ANOVA) evaluating the effects of the interaction between measurement time windows and dynamic time warping-based clusters (MitoStress clusters) on the normalized oxygen consumption rate (OCR) and extracellular acidification rate (ECAR) in equine sperm; Table S5: Output of a mixed-effects linear model evaluating the fixed effects of measurement time (modeled as linear, quadratic, and cubic terms), age in months, and MitoStress clustering on sperm quality traits of stallion sperm. Fixed effects include the intercept, time (first-, second-, and third-degree polynomial terms), age, and MitoStress cluster (with Cluster 1 as the reference group).
