# Supplementary material for: Relationships Between Metabolism of Cryopreserved Equine Sperm Determined by the Seahorse Analyzer and Sperm Characteristics Measured by Flow Cytometry and Computer-Assisted Analysis of Motility
Source: Vet Sci. 2025 Nov 21;12(12):1109. doi: 10.3390/vetsci12121109 (PMC12737643; doi:10.3390/vetsci12121109)
Supplement: Supplementary file 1 [file vetsci-12-01109-s001.zip › Supplemental Figure S1.pdf]

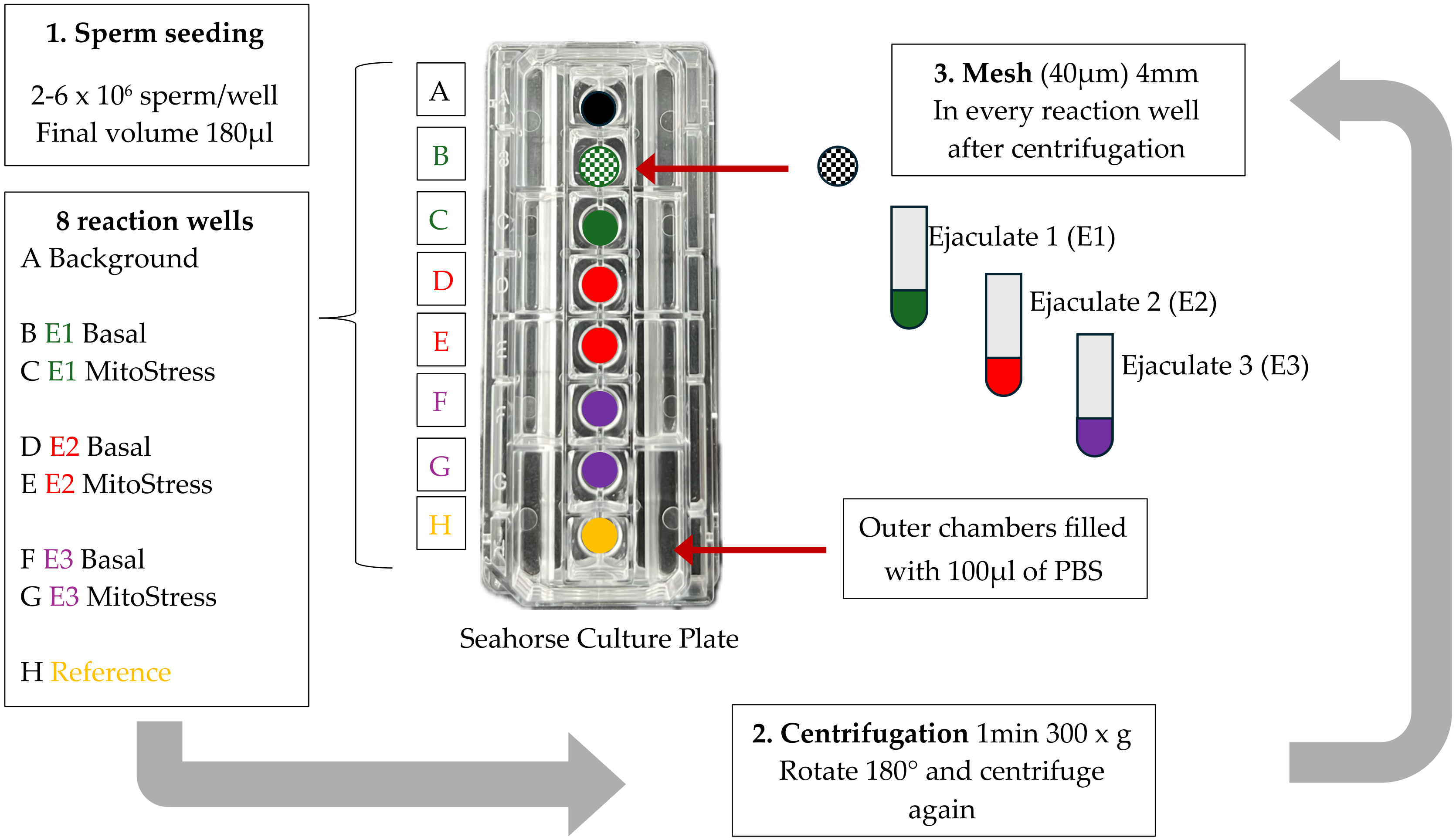

Supplemental Figure S1. Schematic overview of the Seahorse Culture Plate setup and semen seeding procedure prior to Seahorse assay. Each well was coated with 15 µL of concanavalin A (0.5 mg/mL) 18 hours before the assay to facilitate sperm adhesion. Two wells were used for one ejaculate to measure basal OCR and ECAR without injections of mitochondrial reagents and the other well was used to perform the MitoStress Test The coated culture plate was loaded from position B-G with 20µl of raw semen to achieve a concentration of 2-6 x 10^6 sperm per well. Position H was used as reference. The wells were then filled with 160µl of Tyrode's solution to a total volume of 180µl/well. Position A was only filled with 180µl of Tyrode's solution for background measurements. Each of the outer chambers were filled with 100µl of PBS, and the culture plate was centrifuged at 300 x g for 1 min at 23°C, then rotated 180° and centrifuged again for 1 min. Forty µm nylon meshes (saturated in Tyrode's solution) were inserted into each well to keep the sperm at the bottom.
