# Supplementary material for: Relationships Between Metabolism of Cryopreserved Equine Sperm Determined by the Seahorse Analyzer and Sperm Characteristics Measured by Flow Cytometry and Computer-Assisted Analysis of Motility
Source: Vet Sci. 2025 Nov 21;12(12):1109. doi: 10.3390/vetsci12121109 (PMC12737643; doi:10.3390/vetsci12121109)
Supplement: Supplementary file 1 [file vetsci-12-01109-s001.zip › Supplemental Figure S2.pdf]

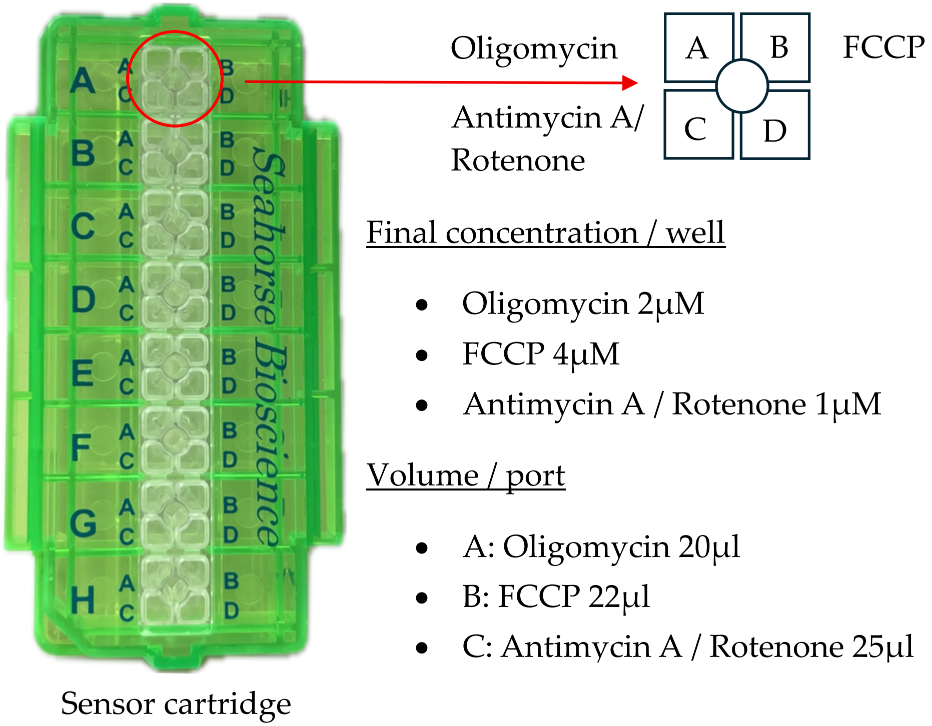

Supplemental Figure S2. Preparations of injections to perform the MitoStress Test. Reagents were prepared and placed in the ports of the sensor cartridge. For the first injection, 20µl of oligomycin working solution was pipetted into port A, resulting in a final concentration of 2µM oligomycin per well. For the FCCP injection, 22µl was added to port B for a final concentration of 4µM FCCP per well. Port C was loaded with 25µl of antimycin A and rotenone working solution, with a final concentration of 1µM of each per well. The ports of the wells without reagent, were filled with the same volumes of Tyrode's solution as negative control.
